# Very Early MoCA Can Predict Functional Dependence at 3 Months After Stroke: A Longitudinal, Cohort Study

**DOI:** 10.3389/fneur.2019.01051

**Published:** 2019-10-11

**Authors:** Tamar Abzhandadze, Lena Rafsten, Åsa Lundgren Nilsson, Annie Palstam, Katharina S. Sunnerhagen

**Affiliations:** ^1^Institute of Neuroscience and Physiology, Rehabilitation Medicine, University of Gothenburg, Gothenburg, Sweden; ^2^Centre for Person-Centred Care (GPCC), University of Gothenburg, Gothenburg, Sweden

**Keywords:** acute stroke, ADL, cognitive function, global disability, modified Rankin Scale, sensitivity, specificity

## Abstract

**Introduction:** After a stroke, cognitive impairment is commonly associated with poor functional outcomes. The primary aim of this study was to investigate if cognitive function, assessed with the Montreal Cognitive Assessment (MoCA) 36–48 h after stroke, could predict functional dependence 3 months later. The secondary aim was to identify an optimal threshold for the MoCA score that could predict functional dependence.

**Materials and Methods:** This was a longitudinal cohort study. The research database from a stroke unit at the Sahlgrenska University Hospital was linked with the Swedish Stroke Register—Riksstroke. Cognitive function and activities of daily living (ADL) were assessed with the MoCA and the Barthel Index (BI), respectively, 36–48 h after stroke. Functional outcome 3 months after stroke was studied with the modified Rankin Scale. The predictive characteristics of the MoCA were investigated using logistic regression analyses. Receiver operating characteristic curves (AUC) were used for identifying the optimal cutoff score on the MoCA for predicting functional dependence. The MoCA score that had equal sensitivity and specificity was chosen as the optimal score for predicting functional dependence.

**Results:** A total of 305 participants were included in the study (mean age: 68.8 years, *n* = 179 men). The MoCA quartiles were a significant predictor of functional dependence 3 months after stroke as an individual variable (*p* < 0.001, AUC = 0.72) and when adjusted for covariates such as age at stroke onset, living arrangement prior to stroke, and ADL measured with BI within 36–48 h after stroke (*p* = 0.01, AUC = 0.84). The MoCA score of ≤23 for impaired cognition had equal sensitivity and specificity for predicting functional dependence 3 months after stroke.

**Discussion and Conclusion:** Cognitive function assessed with the MoCA within 36–48 h after stroke could predict functional dependence 3 months later. The participants with MoCA scores ≤23 for impaired cognition were more likely to be functionally dependent.

## Introduction

The length of hospital stay after stroke has decreased in Sweden (1), from 13 days to 7 days during the last decade (2007–2017). Thus, it has become necessary to identify possible stroke-related impairments early, to predict the need for help in daily living after discharge, and initiate rehabilitation tailored to the patient. Neurological impairment, older age, female sex, and previous stroke are well-known predictors of an increased need for help after stroke (2). Impaired cognition has also been associated with dependence (3, 4).

Cognitive impairment is very common, even after a seemingly successful neurological recovery (5, 6). A first assessment of cognitive function is common clinical practice in acute stroke care units in Sweden. The commonly used assessment instrument, the Montreal Cognitive Assessment (MoCA), is a brief screening tool for evaluation of cognitive function (7). Various MoCA cutoffs have been suggested for the Swedish population depending on age and education (8). Few studies have investigated the relation between cognition assessed with the MoCA and dependence post-stroke. Previous research showed that the MoCA could predict functional dependence at a later stage of stroke. However, in these studies, the MoCA was administered within 7 days to >1 year after stroke (3, 9, 10). Two studies had a small sample size (3, 10), one included people with transient ischemic attack (TIA) (9), and one only included intracerebral hemorrhage (10). Only one study had 3 months of follow-up for functional outcomes (9). Consequently, the predictive characteristics of the MoCA need further examination, especially when it comes to very early assessments.

The primary aim of the study was to investigate if cognitive function assessed with the MoCA 36–48 h after stroke could predict functional outcomes 3 months later; the secondary aim was to identify an optimal score on the MoCA for making the prediction.

## Materials and Methods

### Availability of Data and Material

Complete data cannot be made publicly available for ethical and legal reasons, according to the Swedish regulations (https://etikprovning.se/for-forskare/ansvar/). Researchers can submit requests for data to the authors (contact: ks.sunnerhagen@neuro.gu.se).

### Study Sample

This was a longitudinal cohort study. Participants admitted to a comprehensive stroke unit at Sahlgrenska University Hospital, Gothenburg, Sweden, between 2011 and 2016 were screened and registered in a research database (11). This database was linked with the Swedish Stroke Register—Riksstroke (12). The Riksstroke is a hospital-based register that covers all Swedish hospitals treating stroke. The patient coverage is around 91% (12). Data from acute and 3-month follow-up forms were used. The regional ethical review board in Gothenburg approved the study (042–11, amendment T 966-17).

The inclusion criteria were as follows: age ≥18 years at stroke onset, confirmed stroke diagnosis according to the World Health Organization criteria [a clinical syndrome consisting of rapidly developing clinical signs of focal (or global in case of coma) disturbance of cerebral function lasting more than 24 h or leading to death with no apparent cause other than a vascular origin], the data registered on each task of the MoCA, and independence in activities of daily living (ADL) and mobility prior to stroke according to data from Riksstroke's acute form. The exclusion criterion was participants with subarachnoid hemorrhage.

### Procedure

Cognitive function and ADL performance were assessed by occupational therapists (OTs) within 36–48 h of admission to the stroke unit. The OTs working at the stroke units have attended workshops on a regular basis regarding cognitive functions after stroke. They have also participated in peer discussions about the MoCA assessments as well as scoring. The information about neurological status upon admittance to the hospital was extracted from medical records. The data in the Riksstroke acute form was registered by staff at the stroke care unit. Postal, self-administered questionnaires were used for gathering 3-month follow-up data. The nurse telephoned the participants if the questionnaire was not returned within 1 month after dispatching a reminder letter.

### Outcomes

#### Dependent Variable

The modified Rankin Scale (mRS) was estimated from the Riksstroke's five self-reported questions at 3 months, according to Eriksson et al. (13). The questions were as follows: Are you today dependent on a family member/next-of-kin for help/support? Where are you staying now? How is your mobility now? Do you need help from someone to visit the toilet? Do you need help getting dressed and undressed? The translation algorithm of the mRS scores is presented in Supplemental Table I. The mRS score range is from 0 to 6, where 0 indicates no symptoms and 6 indicates death.

#### Independent Variables

Cognitive function was assessed with the MoCA (7). The score range is 0 to 30; the threshold for normal cognitive function is ≥26 (7). The MoCA has six cognitive domains: short-term memory, visuospatial abilities, executive functions, attention and working memory, language, and orientation to time and space (Supplemental Table II) (7). The ADL performance was assessed with the Barthel Index (BI) (14). The score range on the BI is 0–100, with lower scores indicating more dependence. Both the MoCA and BI were assessed 36–48 h post-stroke. Stroke-related neurological deficits at the admittance to the hospital were estimated with the National Institutes of Health Stroke Scale (NIHSS) (15), with a score range of 0 to 42. The stroke was classified according to the Oxfordshire Community Stroke Project Classification (OCSP) (16). Furthermore, the data about ADL and mobility prior to stroke, comorbidities, accommodations, length of hospital stay, discharge destination, stroke risk factors, and reperfusion treatment were extracted from the acute form of the Riksstroke. The data about living arrangements after stroke were extracted from the 3-month follow-up form from the Riksstroke.

### Statistics

The data were analyzed with IBM SPSS Statistics 25.0 (IBM SPSS Statistics for Windows, Version 25.0. Armonk, NY: IBM Corp). The tests were two-sided and conducted at the 5% significance level. The characteristics of the study participants are presented with descriptive statistics. Group comparisons were made with Pearson's χ^2^ test and the Mann–Whitney *U-*test. The effect size of the Mann–Whitney *U*-test was calculated according to equation: $r^{2}=\frac{Z^{2}}{N}$, *r*^2^ under 0.2 was regarded as small (17).

#### Logistic Regression Analyses

The mRS scores were dichotomized: 0–2 were coded as functional independence “0” and scores ≥3 were coded as functional dependence “1.” The total scores of the MoCA, NIHSS, and BI were recoded into quartiles (Supplemental Table III). Nineteen possible independent variables were identified from previous studies (2, 3, 9, 18, 19) and clinical experience (Supplemental Table III and Supplemental Figure). They were further tested for collinearity with Spearman's rank correlation test (*r*_s_). Variables with *r*_s_ < ±0.7 were not regarded as having collinearity. Logistic regressions with the purposeful selection (PS) of variables were performed (20). The PS comprises several steps for analyses of independent variables, and each step of PS has different cutoffs for *p*-value (Supplemental Figure). According to the method, although some variables do not achieve statistical significance, they can be entered into the final regression model due to their clinical importance (20). In the current study, 19 independent variables were analyzed (Supplemental Figure, step 1). Of those, the final regression model included the MoCA as the main predictor and three other variables that reached statistical significance: age at stroke onset, living arrangement prior to stroke, and ADL measured with BI within 36–48 h after stroke (Supplemental Figure). Furthermore, to understand if the cognitive domains of the MoCA could predict functional dependence 3 months after stroke, each cognitive domain was pooled in the adjusted final model, one at a time. Clinically important variables (NIHSS, sex, and previous stroke) that did not reach statistical significance were also pooled into the final model, one at a time. The overall performance of the models has not changed, which means that clinically important variables could not contribute to the prediction of functional dependence 3 months after stroke.

The cutoff score from the MoCA for predicting functional dependence was studied with receiver operating characteristic (ROC) curves. In the beginning, the total MoCA score and dichotomized mRS were entered into the analyses. Several cutoff values for impaired cognition were chosen. Furthermore, the sensitivity, specificity, positive predictive value (PPV), negative predictive value (NPV), and the Youden *J* index were calculated based on dichotomized MoCA and mRS scores.

## Results

### Study Participants

**Figure 1:**
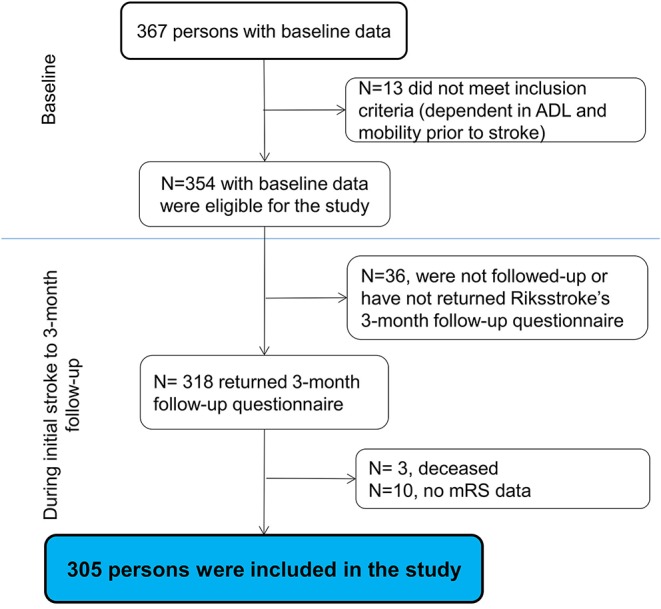
The flowchart of the study participants (ADL, activities of daily living; mRS, modified Rankin Scale).

In total, 354 participants with baseline data met the inclusion criteria. The 3-month follow-up questionnaire was returned by 89.8% (*n* = 318). The participants who returned the questionnaire (*n* = 318) had significantly lower admission NIHSS scores (*p* = 0.04) compared with the participants who did not return (*n* = 36) the questionnaire. The differences regarding sex (*p* = 0.71) and age (*p* = 0.14) were not significant. Among the returned questionnaires (*n* = 318), three participants were reported deceased by their next of kin and 10 did not have mRS data; thus, they were excluded, yielding 305 participants (Figure 1).

Of 305 participants, 126 were female, and the mean age was 69 years at stroke onset (Table 1). Participants aged ≤65 years scored significantly higher than participants aged ≥66 years on the BI and the MoCA, and they had a shorter hospital stay (Table 2).

**Table 1:** Characteristics of the study participants (*n* = 305).

**Baseline characteristics**	
Sex, female, *n* (%)	126 (41%)
Age, years, mean (SD)	68.8 (14.5)
*Accommodation prior to stroke, n (%):*	
Own accommodation without home help	286 (94)
*Household prior stroke, n (%)*	
Living alone	121 (40)
Shared household	184 (60)
*Risk factors/comorbidities, yes, n (%):*	
Diabetes	38 (12)
Current smoker	43 (15)
Previous stroke	51 (17)
Treated for hypertension at onset of stroke	148 (50)
*Stroke type and classification, n (%):*	
Ischemic stroke:	276 (90)
Total anterior circulation stroke	3 (1)
Partial anterior circulation stroke	47 (15)
Anterior circulation syndrome	97 (32)
Lacunar syndrome	129 (42)
Hemorrhage	29 (10)
*Reperfusion*, n (%):	69 (23)
*Stroke-related outcomes*, median (range)*:*	
The Barthel Index^*^	95 (10–100)
The Montreal Cognitive Assessment^†^	24 (3–30)
NIHSS^‡^	2 (0–19)
Length of hospital stay	6 (0^§^-37)
*Discharge destination from the stroke unit, n (%)*	
Own accommodation	272 (89)
Arranged accommodation	10 (3)
Other acute clinic	4 (1)
Geriatric/rehab unit	19 (6)
**Three months after stroke**	
*Accommodation after stroke, n (%):*	
Own accommodation without home help	250 (83)
Own accommodation/arranged accommodation with help	47 (15)
Other	5 (2)
*Household, n (%)*	
Living alone	104 (35)
Shared household	194 (65)

* The Barthel Index and

† *the Montreal Cognitive Assessment assessed 36–48 h after stroke*.

‡ *NIHSS, The National Institute of Health Stroke Scale admission score*.

§ *0 refers to ≤24 h of hospital stay. The sum may be different due to missing values. Variables with missing data, n (%): for baseline data—diabetes 7 (2.3%), previous stroke 7 (2.3%), treated for hypertension at the onset of stroke 7 (2.3%), and reperfusion 11 (3.6%). For 3-month follow-up—accommodation 3 (1%) and household 7 (2.3%)*.

**Table 2:** Baseline characteristics of study participants stratified by age at onset of stroke.

**Characteristics**	**≤65 years, *n* = 108**	**≥66 years, *n* = 197**	***p*-value**
*Sex, n (%)*			0.69^*^
Female	43 (40)	83 (42)	
Male	65 (60)	114 (58)	
*Accommodation prior to stroke, n (%):*			0.005^*^
Own accommodation without home help	107 (99)	179 (91)	
Household, prior to stroke, *n (%):*			0.05^*^
Lives alone	35 (32)	86 (44)	
Shared household	73 (68)	111 (56)	
*Risk factors/comorbidities, yes, n (%):*			
Diabetes	8 (8)	30 (15)	0.05^*^
Current smoker	24 (22)	9 (10)	0.003^*^
Previous stroke	12 (11)	39 (20)	0.05^*^
Treated for hypertension at onset of stroke	36 (34)	112 (58)	0.000^*^
*Stroke type and classification, n (%):*			0.217^†^
Ischemic stroke:	94 (87)	182 (92)	
Total anterior circulation stroke	1 (1)	2 (1)	
Partial anterior circulation stroke	9 (8)	38 (19)	
Posterior circulation syndrome	42 (39)	55 (28)	
Lacunar syndrome	42 (39)	87 (44)	
Hemorrhage	14 (13)	15 (8)	
Reperfusion, *n (%):*	23 (22)	46 (24)	0.73^*^
*Stroke-related outcomes, median (range):*			
The Barthel Index^‡^	100 (35–100)	90 (10–100)	0.000^†^
The MoCA^‡^	28 (16–30)	22 (3–30)	0.000^†^
NIHSS^§^	1 (0–14)	2 (0–19)	0.04^†^
Length of hospital stay	5 (1–37)	7 (0–33)	0.02^†^
*Discharge destination from the stroke unit, n (%)*			0.56^†^
Own accommodation	95 (88)	177 (90)	
Arranged accommodation	2 (2)	8 (4)	
Other acute clinic	2 (2)	2 (1)	
Geriatric/rehab unit	9 (8)	10 (5)	

* *Pearson's χ^2^ test, two tailed*.

† *Mann–Whitney U-test, two tailed*.

‡ *The Barthel index and the MoCA-the Montreal Cognitive Assessment: assessed 36–48 h post stroke*.

§ *NIHSS-the National Institute of Health Stroke Scale—assessed at stroke onset. The sum may be different due to missing values. Variables with missing data n (%): participants ≤65 years—diabetes 4 (3.7%), treated for hypertension at onset of stroke 3 (2.8%), previous stroke 3 (2.8%), and reperfusion 5 (4.6%). Participants ≥66 years—diabetes 3 (1.5%), treated for hypertension at onset of stroke 4 (2%), previous stroke 4 (2%), and reperfusion 6 (3%)*.

**Figure 2:**
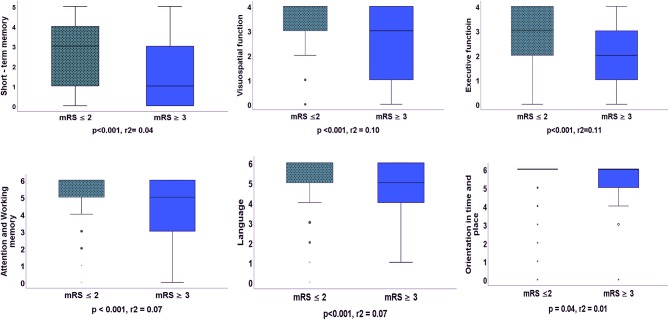
Difference between the cognitive domains of the Montreal Cognitive Assessment administered 36–48 h after stroke for participants with functional independence (mRS ≤2) and functional dependence (mRS ≥3) 3 months after stroke. Statistics: Mann–Whitney *U*-test, *r*^2^, the effect size of the Mann–Whitney *U*-test.

Three months after stroke, 29% of the participants were functionally dependent (mRS ≥3), and 83% were living in their own accommodations without help from community services. Admission scores on BI (*p* < 0.001, *r*^2^ = 0.19) and the MoCA (*p* < 0.001, *r*^2^ = 0.13) were significantly lower in the participants with a mRS ≥3 compared with participants with a mRS ≤2. Furthermore, admission score on the NIHSS (*p* = 0.006, *r*^2^ = 0.02) was significantly higher in the participants with a mRS ≥3 compared with participants with a mRS ≤2. Participants with a mRS ≥3 also scored significantly lower on all cognitive domains of the baseline MoCA compared with the participants with a mRS ≤2 (Figure 2).

### MoCA's Predictive Characteristics for Functional Dependence 3 Months After Stroke

The selection process of the independent variables is presented in Supplemental Table III and the Supplemental Figure. There was no collinearity between independent variables. Cognitive function assessed with the MoCA within 36–48 h after stroke predicted functional dependence 3 months later as a single variable and when adjusted for covariates (Table 3, Supplemental Table IV). Furthermore, impaired visuospatial functions (OR = 0.75, 95% CI 0.58–0.98), executive functions (OR = 0.65, 95% CI 0.50–0.81), and language (OR = 0.73, 95% CI 0.56–0.94) could predict functional dependence 3 months later (Table 3). The overall predictive value of the final model with the MoCA did not change when regression analyses included clinically significant variables such as previous stroke, sex, and admission NIHSS.

**Table 3:** Predictive characteristics from the Montreal Cognitive Assessment and its cognitive domains for functional dependence (mRS ≥ 3) 3 months after stroke.

**Model#**	**Independent Variables**	OR^*^ (95% CI^†^)	***p*-value**	**AUC^‡^**	**Nagelkerke *R*^**2**^**	**H–L^§^ test**
**1**^**||**^	MoCA^#^, Q_1_, ref.		<0.001	0.72	0.19	1.00
	MoCA, Q_2_	0.35 (0.20–0.63)	<0.001			
	MoCA, Q_3_	0.08 (0.03–0.19)	<0.001			
2^**^	MoCA^#^, Q_1_, ref.		0.01	0.84	0.41	0.41
	MoCA, Q_2_	0.60 (0.31–1.17)	0.14			
	MoCA, Q_3_	0.22 (0.08–0.58)	0.002			
3^**^	Short-term memory^†^^†^	0.95 (0.78–1.15)	0.58	0.82	0.39	0.48
4^**^	Visuospatial functions^†^^†^	0.75 (0.58–0.98)	0.03	0.83	0.39	0.84
5^**^	Executive functions^†^^†^	0.65 (0.50–0.81)	<0.001	0.83	0.41	0.74
6^**^	Attention and working memory^†^^†^	0.81 (0.65–1.01)	0.07	0.82	0.39	0.90
7^**^	Language^†^^†^	0.73 (0.56–0.94)	0.02	0.83	0.40	0.26
8^**^	Orientation, time, and space^†^^†^	0.99 (0.69–1.42)	0.97	0.82	0.38	0.63

***Bold** model indicates statistical significance*.

* OR, odds ratio;

† 95%CI, 95% confidence interval;

‡ AUC, area under a receiver operating characteristic curve;

§ *H–L test, the Hosmer–Lemeshow test. ^||^Unadjusted model*.

** *Models adjusted for age at stroke onset, living arrangement prior to stroke, activities of daily living measured with the Barthel Index within 36–48 h after stroke. #MoCA—Montreal Cognitive Assessment: Q_1_–first quartile, 21 points; Q_2_–second quartile, 24 points; and Q_3_–third quartile, 27 points. Cognitive domains of MoCA: short-term memory, range 0–5 points; visuospatial functions, range 0–4 points; executive functions, range 0–4 points; attention and working memory, range 0–6 points; language, range 0–6 points; orientation time and space, range 0–6 points*.

### The Cutoff Score of the MoCA for Predicting Functional Dependence

**Figure 3:**
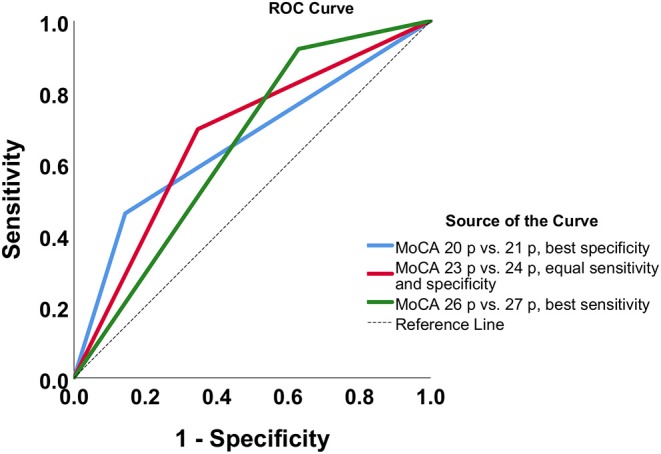
The receiver operating characteristic (ROC) curve for dichotomized modified Rankin Scale scores and cutoff values for the Montreal Cognitive Assessment (MoCA); p refers to points on the MoCA.

Seven possible cutoff scores of the MoCA were examined (Table 4). The MoCA cutoff of ≤23 points for impaired cognitive function had equally good sensitivity (69.7%) and specificity (65.3%). Thus, it was chosen as the most optimal cutoff score for predicting functional dependence 3 months after stroke. The ROC curves are presented in Figure 3.

**Table 4:** Examination of the thresholds on the Montreal Cognitive Assessment for the direction of functional dependence (mRS ≥ 3) 3 months after stroke.

MoCA^*^ Cutoff	**Sensitivity % (95%CI)**	**Specificity % (95%CI)**	**PPV^†^ % (95%CI)**	**NPV^‡^% (95%CI)**	**AUC^§^**	**YI^§^**
**20 vs. 21**	46.1 (35.4–56.9)	**85.6 (80.2**–**90.0)**	56.9 (47.1–66.3)	79.4 (75.9–82.5)	0.66	0.32
21 vs. 22	50.6 (39.7–61.3)	81.0 (75.1–86.0)	52.3 (43.8–60.7)	79.9 (76.1–83.2)	0.66	0.32
22 vs. 23	60.7 (49.7–70.9)	73.6 (67.2–79.4)	48.6 (41.7–55.6)	82.0 (77.6–85.6)	0.67	0.35
**23 vs. 24**	**69.7 (59.0**–**79.0)**	**65.3 (58.5**–**71.6)**	45.3 (39.7–50.9)	83.9 (78.9–87.8)	0.67	0.35
24 vs. 25	75.3 (65.0–83.8)	57.4 (50.5–64.1)	42.1 (37.5–46.9)	84.9 (79.4–89.2)	0.66	0.32
25 vs. 26	82.0 (72.4–89.4)	47.2 (40.4–54.1)	39.0 (35.3–43.0)	86.4 (80.0–91.0)	0.65	0.29
**26 vs. 27**	**92.1 (84.5**–**96.8)**	37.0 (30.6–43.8)	37.6 (34.9–40.4)	91.9 (84.6–96.0)	0.65	0.29

*95%CI, 95% confidence intervals*.

* *MoCA, Montreal Cognitive Assessment*.

† *PPV, positive predictive value*,

‡ *NPV, negative predictive value,^‡^AUC, area under a receiver operating characteristic curve*,

§ *YI, Youden's index. Bold values indicate high sensitivity, equally high sensitivity and specificity, high specificity*.

## Discussion

This longitudinal cohort study showed that cognitive screening performed with the MoCA, within 36–48 h after stroke, could predict functional dependence 3 months later, independently as well as when adjusted for covariates. Moreover, the predictive significance of the MoCA remained when the clinically significant variables were pooled into the final model. This is a clinically important finding, highlighting the efficacy of cognitive assessment with the MoCA early after stroke for predicting the need of help 3 months later. Our results are partly in line with previous studies (3, 9) reporting that the baseline cognitive screening with the MoCA can predict functional dependence 3–6 months after stroke (3, 9). Furthermore, in one study, the predictive value of the MoCA was smaller than the NIHSS in the population with NIHSS scores >2 points (9). In the current study, however, the NIHSS score did not reach significance. It is possible that in the population with mild stroke (NIHSS ≤2 points), the MoCA is a more sensitive predictor of functional dependence. The cognitive deficits are persistent after seemingly successful neurological recovery (6). Moreover, people with stroke can have impaired cognition several months after stroke (21); thus, their functional outcomes can be affected negatively. Theoretically, there can be a positive correlation between different levels of mRS and cognitive resources; however, this important question could not be investigated in this study. Furthermore, the study participants had a high median age in the current study. The greater age is associated with lower scores on the MoCA (8) and deterioration of functional outcomes after stroke (22). Thus, age should be considered when interpreting the MoCA's predictive value for functional dependence 3 months after stroke.

Participants with a mRS ≥3 scored significantly lower on all cognitive domains of the MoCA compared with the participants with a mRS ≤2 in the current study. Furthermore, visuospatial functions, executive functions, and language were significant predictors for functional dependence 3 months after stroke. Executive function is multimodal cognitive domain comprising inhibition, interference control, working memory, and cognitive flexibility (23). The executive functions seem to be involved in visuospatial functions and can influence not only visuospatial working memory but also verbal working memory (24). The results of the present study fits with previous studies (3, 25–27) showing the importance of these cognitive domains for prediction of functional outcomes. However, the MoCA's cognitive domains seem to be less sensitive in determining domain-specific cognitive difficulties (28). Thus, we recommend using the total score of the MoCA rather than the scores on separate cognitive domains.

A MoCA score ≤23 points for impaired cognitive functioning had equal sensitivity and specificity for predicting functional dependence; thus, it was chosen as the optimal cutoff value. Decision about taking equal sensitivity and specificity for choosing the cutoff was made for achieving the balance between the probability of the MoCA to identify cognitive deficits if the participants had it and the probability of the MoCA not to identify cognitive deficits if the participants did not have it. Cognitive performance very early after stroke can be affected by many factors such as tiredness, stroke-related crisis, medications, etc. Thus, it is important to be aware that very early cognitive assessment could be influenced by these temporary circumstances. Furthermore, when equal sensitivity and specificity is taken as the major diagnostic measurement for the cutoff value, it will affect the false-positive and false-negative rate. However, the aim of the study was to identify the cutoff value on MoCA, which could predict functional dependence 3 months later; thus, these measurements were not taken into consideration when choosing the cutoff value.

There is no concordance regarding the optimal cutoff score of the MoCA for predicting functional dependence. The MoCA scores of <20 points (27), <21 points (29), and <26 points (3) for impaired cognition were previously associated with functional dependence. In these studies, optimal cutoff score for prediction (3, 27, 29) were identified by taking sensitivity and specificity as equally important. These different results could be explained by several factors. It is known that the MoCA results can be affected by age and education (8). More studies on a larger stroke population have to be performed to investigate possible MoCA cutoffs for predicting functional outcomes at a later stage of stroke.

There are several limitations of the study. The dropout rate between baseline and the 3-month follow-up was 10%, and participants with more severe stroke were lost. The mRS was calculated according to five questions from the Riksstroke. The agreement algorithm between mRS grades and Riksstroke's questions was developed by Eriksson et al. (13) and since then has been used in the Riksstroke register-based studies. However, for avoiding the methodological issues previously described (13), dichotomized coding of variables was performed from the beginning. It is possible that this direct dichotomization affected the results, especially when groups with mRS scores ≤2 and ≥3 were compared (30). Another limitation is that there is no information about the participants' education and cognitive status prior to the stroke. Some participants were old, thus there is a probability that they might have impaired cognition related to high age. However, these participants were living independently without help. Participants in working age were productive (31). Furthermore, the participants could not be controlled for brief confusion shortly after the onset of stroke.

The study has several strengths. The study sample is representative of patients seen in clinical practice; thus, the results have ecological validity. The novelty of the study is that cognitive function was evaluated with the MoCA within 36–48 h after stroke in a population with mild to moderate stroke. The MoCA is applicable in the acute stroke setting in participants with mild to moderate stroke (32). Although the study sample had a wide range of neurological deficits—NIHSS 0–19, the majority of the study participants had a mild to moderate stroke at admission to the stroke unit. Our sample is representative compared to the Swedish stroke population, where the majority has mild to moderate stroke at the admittance to the hospital. The assessment instruments used in the study, as well as Riksstroke's questionnaire, are valid for the stroke population. The sample size is relatively large and comprises participants with a verified stroke diagnosis and a wide age range. Accordingly, the study results showing the MoCA's very early predictive value for functional dependence 3 months after a stroke could be generalized to the population with mild stroke, but this needs to be explored in a larger sample.

## Conclusion

In the current study, the MoCA could partly predict functional dependence 3 months after stroke in a population with mild to moderate stroke. This is a clinically important finding, which can strengthen the recommendation for cognitive screening (33). The results could be helpful for health care practitioners in stroke units to start early communication with people who had a stroke and their relatives about the possible effects of impaired cognition on everyday activities. However, it is still unclear if very early cognitive screening with the MoCA can predict the discharge destination.

The health care professionals could use the MoCA thresholds presented in this study in their clinical practice. People with a MoCA score ≤23 for impaired cognition are more likely to demonstrate functional dependence 3 months after stroke. Thus, this group might benefit from in-depth activity and cognitive assessments before discharge from the hospital and structured follow-up after discharge. In contrast, people with a MoCA score ≥24 (normal cognitive functioning) are less likely to be functionally dependent after discharge from the hospital. However, a systematic follow-up after discharge from the hospital is strongly recommended. Further research should evaluate normative data for the ability of the MoCA cutoff to predict functional dependence in the Swedish stroke population.

## Data Availability Statement

According to the Swedish regulations (https://etikprovning.se/for-forskare/ansvar/) the datasets generated for this study cannot be made publicly available for ethical and legal reasons. Researchers can submit requests for data to the authors (contact: ks.sunnerhagen@neuro.gu.se).

## Ethics Statement

This study involves human participants. The study was reviewed and approved by the regional ethical review board in Gothenburg (042–11, amendment T 966-17). Written informed consent for participation was not required for this study in accordance with the national legislation and the institutional requirements.

## Author Contributions

TA: acquisition of data, conceptualization of the study, analysis and interpretation of the data and drafting of the manuscript. LR: acquisition of data, design or conceptualization of the study, and revising the manuscript for intellectual content. ÅL: design or conceptualization of the study and revising the manuscript for intellectual content. AP: interpretation of the data and revising the manuscript for intellectual content. KS: design or conceptualization of the study, interpretation of the data, and revising the manuscript for intellectual content.

### Conflict of Interest

The authors declare that the research was conducted in the absence of any commercial or financial relationships that could be construed as a potential conflict of interest.
