# Supplementary material for: Very Early MoCA Can Predict Functional Dependence at 3 Months After Stroke: A Longitudinal, Cohort Study
Source: Front Neurol. 2019 Oct 11;10:1051. doi: 10.3389/fneur.2019.01051 (PMC6798188; doi:10.3389/fneur.2019.01051)
Supplement: Supplementary file 1 [file Data_Sheet_1.pdf]

Supplemental Material

Very early MoCA can predict functional dependence at 3 months after stroke:

A longitudinal, cohort study.

Authors:

Tamar Abzhandadze, MS[1]

Lena Rafsten, MS[1,2]

Åsa Lundgren Nilsson, PhD[1, 2]

Annie Palstam, PhD[1]

Katharina S. Sunnerhagen, MD, PhD[1, 2]

[1] Institute of Neuroscience and Physiology, Rehabilitation medicine, University of Gothenburg. Gothenburg, Sweden. Per Dubbsgatan 14, fl. 3, 413 45 Gothenburg, Sweden.

[2] Centre for Person-Centred Care (GPCC), University of Gothenburg. Gothenburg, Sweden.

Corresponding author: Tamar Abzhandadze. Address: Institute of Neuroscience and Physiology, Rehabilitation medicine, University of Gothenburg, Gothenburg, Sweden.
Per Dubbsgatan 14, fl. 3, 413 45 Gothenburg, Sweden. Fax number: +46 317412659, telephone number: +46 702622897, e-mail address: tamar.abzhandadze@gu.se.

Supplemental tables:

Table I. The algorithm for estimating mRS scores according to Riksstroke's five self-reported questions (adapted from Eriksson et al. [1]).

mRS grade	Definition of adjusted translation, using Riksstroke questions	Answer codes
0-2	Q1: not or only partly dependent on next of kin for help/support.	1 or 3
	Q2: living in own home without community support.	1
	Q3: can move around without help both indoors and outdoors.	1
	Q4: can manage toilet visits without assistance.	1
	Q5: can manage to dress/undress without help.	1
3	Q1: entirely dependent on next of kin for help/support or	2,
	Q2: not living in own home without community support or	2, 3, 5, 6, 7
	Q3: cannot move around without help outdoors or	2
	Q3: can move around without help at least indoors	1 or 2
	Q4: can manage toilet visits without assistance	1
	Q5: can manage to dress/undress without help	1
4	Q3: cannot move around without help indoors or	3
	Q4: need help to go to the toilet or	2
	Q5: need help dressing/undressing	2
5	Q2: not living at home	3, 5, 6, 7
	Q3: need another person’s help to move	3
	Q4: need help to go to the toilet	2
	Q5: need help dressing/undressing	2

Q1: Are you today dependent on a family member/next-of-kin for help/support (1- partly dependent, 2- entirely dependent, 3 - no, not at all)?
Q2: Where are you staying now (1- in own home without community support, 2 - in own home with community support, 3- living in community facility, 5- in acute care hospital, 6 - other, 7 - in a geriatric/rehabilitation unit)?
Q3: How mobile are you (1 - I can move around without help both indoors and outdoors, 2 - I can move around without help indoors but not outdoors, 3 - I need another person’s help to move)?
Q4: Do you receive help from anybody to go to the toilet (1 - I can manage toilet visits without assistance, 2- I need help to go to the toilet)?
Q5: Do you receive help with dressing/undressing (1 - I can manage dress/undress without help, 2- I need help dressing/undressing)?

Table II. The algorithm for calculating the six cognitive domains of the Montreal Cognitive Assessment[2].

Cognitive domain on Montreal Cognitive Assessment, (total score)	Task	Task specific score
Short term memory, (5)	The short-term memory recall task involves two learning trials of five nouns and delayed recall after approximately 5 min.	5
Visuospatial abilities, (4)	A clock-drawing task	3
	A three-dimensional cube copy	1
Executive functions, (4)	An alternation task adapted from the Trail Making B task	1
	A phonemic fluency task	1
	A two-item verbal abstraction task	2
Attention, concentration, and working memory, (6)	A sustained attention task (target detection using tapping)	1
	A serial subtraction task	3
	Digits forward and backward	2
Language, (6)	A three-item confrontation naming task with low-familiarity animals	3
	Repetition of two syntactically complex sentences	2
	The aforementioned fluency task	1
Orientation to time and place, (6)	Date	1
	Month	1
	Year	1
	Day	1
	Place	1
	City	1

Table III. Independent variables entered into univariate logistic analysis.

Variable	Description
Household, prior to stroke	0 – Yes, lives alone, 1 – No, shares household
Accommodation, prior to stroke	0 – Own accommodation without home help<br>1 – Own accommodation/arranged accommodation with help
Previous stroke	0 – No, 1 – Yes
Diabetes	0 – No, 1 – Yes
Age at stroke onset	Scale, range 19-97 years
Sex	1 – Men, 2 – Women
Length of hospital stay	Scale, range 0-45 days
Paresis, upper limb, right	0 – No drift, limb holds for 90 (or 45) degrees for full 10 s.<br>1 – Drift, limb holds for 90 (or 45) degrees, but drifts down before full 10 s, but does not hit bed/other support.<br>2 – Some effort against gravity, limb cannot get to or maintain (if cued) 90 (or 45) degrees, drifts down to bed but has some effort against gravity.<br>3 – No effort against gravity, limb falls.<br>4 – No movement.
Paresis, upper limb, left	0 – No drift, limb holds for 90 (or 45) degrees for full 10 s.<br>1 – Drift, limb holds for 90 (or 45) degrees, but drifts down before full 10 s, but does not hit bed/other support.<br>2 – Some effort against gravity, limb cannot get to or maintain (if cued) 90 (or 45) degrees, drifts down to bed but has some effort against gravity.<br>3 – No effort against gravity, limb falls.<br>4 – No movement.
Discharge destination from acute stroke unit	1 – Own accommodation<br>2 – Arranged accommodation<br>3 – Other acute clinic<br>4 – Geriatric/rehab unit
NIHSS[†] at stroke onset, score range 0-19 points.	Quartiles:<br>Q_1 - 0<br>Q_2 - 2<br>Q_3 - 4
BI [‡] assessed 36-48 h post stroke, score range 10-100 points.	Quartiles:<br>Q_1 - 80<br>Q_2 - 95<br>Q_3 - 100
MoCA[§] assessed 36-48 h post stroke, the score range 3-30 points.	Quartiles:<br>Q_1 - 21<br>Q_2 - 24<br>Q_3 - 27
MoCA, short-term memory	Total score was used, range 0 – 5 points
MoCA, visuospatial functions	Total score was used, range 0 – 4 points
MoCA, executive functions	Total score was used, range 0 – 4 points

MoCA, attention and working memory	Total score was used, range 0 – 6 points
MoCA, language	Total score was used, range 0 – 6 points
MoCA, orientation in time and space	Total score was used, range 0 – 6 points

Abbreviations: [†]NIHSS – National Institute of Health Stroke Scale, [‡]BI – Barthel Index, [§]MoCA – Montreal Cognitive Assessment. Note: The total scores of the NIHSS, BI, and MoCA are categorized according to quartiles.

Table IV. Predictive characteristics of the total score of the Montreal Cognitive Assessment (MoCA) for functional dependence (mRS≥3) three months after stroke.

Model	Independent Variables	OR* (95 %CI†)	P-value	AUC‡	Nagelkerke R^2
**1**‖	MoCA#	0.85 (0.80-0.90)	<0.001	0.73	0.15
**2****	MoCA	0.92 (0.87-0.99)	<0.05	0.83	0.39

*OR- odds ratio, † 95%CI - 95% confidence interval, ‡ AUC – area under a receiver operating characteristic curve. ‖Unadjusted model. **Model adjusted for age at stroke onset, living arrangement prior to stroke, activities of daily living measured with the Barthel Index within 36-48 h after stroke. #MoCA – Montreal cognitive Assessment, score range 0-30 points.

Supplemental figure: Process for selecting independent variables for predicting functional dependence 3 months after stroke, n=305.

Step 1. Univariable analyses, variables with p≥0.25 (red text) are eliminated.	Step 2. Multivariable analyses with Wald: backward statistics. Variables with p≥0.05 (red text) are eliminated	Step 3. Multivariable analysis only based on variables with p ≤ 0.05. Variable in red was not significant (p>0.05).	**FINAL Model: multivariable regression analyses model.**	Clinically important variables added to the FINAL model, one at the time.
Household: alone/shared household	Household: alone/shared household	Household: alone/shared household		
Accommodation	Accommodation	Accommodation	**Accommodation**	
Previous stroke	Previous stroke			Previous stroke
Diabetes	Diabetes			
Age	Age	Age	**Age**	
Sex				Sex
Length of hospital stay	Length of hospital stay			
Paresis UL, right				
Paresis UL, left	Paresis UL, left			
Discharge destination from acute stroke unit	Discharge destination from acute stroke unit			
NIHSS	NIHSS			NIHSS
BI	BI	BI	**BI**	
MoCA	MoCA	MoCA	**MoCA**	
MoCA - Short term memory				MoCA - Short term memory
MoCA - Visuospatial Function				MoCA - Visuospatial Function
MoCA - Executive Function				MoCA - Executive Function
MoCA - Attention and Working memory				MoCA - Attention and Working memory
MoCA - Language				MoCA - Language
MoCA - Orientation in time and place				MoCA - Orientation in time and place

Abbreviations: UL - upper limb, NIHSS - the National Institute of Health Stroke Scale, BI - Barthel index, MOCA - Montreal Cognitive Assessment.

References

1. Eriksson M, Appelros P, Norrving B, Terént A, Stegmayr B. Assessment of Functional Outcome in a National Quality Register for Acute Stroke: Can Simple Self-Reported Items Be Transformed Into the Modified Rankin Scale? Stroke. 2007; 38:1384-1386.

2. Nasreddine ZS, Phillips NA, Bédirian V, Charbonneau S, Whitehead V, Collin I, et al. The Montreal Cognitive Assessment, MoCA: A brief screening tool for mild cognitive impairment. J Am Geriatr Soc. 2005; 53:695-699.
